# Isolation and identification of a new strain of nervous necrosis virus from the big-belly seahorse *Hippocampus abdominalis*

**DOI:** 10.1186/s12985-022-01837-8

**Published:** 2022-06-27

**Authors:** Xinxin Chen, Jianfei Qi, Libin He, Huiyu Luo, Jinbo Lin, Fengyan Qiu, Qing Wang, Leyun Zheng

**Affiliations:** 1 Xiamen Xiaodeng Fisheries Science and Technology Co., LTD, Xiamen, 361006 China; 2 Fisheries Research Institute of Fujian, Xiamen, 361000 China; 3 College of Marine Sciences, South China Agricultural University, Guangzhou, 510642 China; 4 Guangdong Laboratory for Lingnan Modern Agriculture, Guangzhou, 510642 China

**Keywords:** Nervous necrosis virus, Big-belly seahorse, Identification, Pathogenicity

## Abstract

**Background:**

*Betanodaviruses*, members of the *Nodaviridae* family, are the causative agents of viral nervous necrosis in fish, resulting in great economic losses worldwide.

**Methods:**

In this study, we isolated a virus strain named seahorse nervous necrosis virus (SHNNV) from cultured big-belly seahorses *Hippocampus abdominalis* in Xiamen city, Fujian Province, China. Virus isolation, PCR detection, phylogenetic analysis, qRT-PCR, fluorescence in situ hybridization and histology were used for virus identification and analysis of virus histopathology. Furthermore, an artificial infection experiment was conducted for virulence testing.

**Results:**

Brain and eye tissue homogenates of diseased big-belly seahorses were inoculated onto a grouper spleen (GS) cell monolayer at 28 °C. Tissue homogenates induced obvious cytopathic effects in GS cells. PCR and sequencing analyses revealed that the virus belonged to *Betanodavirus* and shared high sequence identity with red-spotted grouper nervous necrosis virus isolates. qRT-PCR and fluorescence in situ hybridization revealed that SHNNV mainly attacked the brain and eye. Histopathological examination revealed that the virus led to cytoplasmic vacuolation in the brain and retinal tissues. Infection experiments confirmed that SHNNV was highly infectious, causing massive death in big-belly seahorses.

**Conclusion:**

A novel seahorse *betanodavirus* from the big-belly seahorse cultured in China was discovered. This finding will contribute to the development of efficient strategies for disease management in aquaculture.

**Supplementary Information:**

The online version contains supplementary material available at 10.1186/s12985-022-01837-8.

## Background

Seahorses are a group of aquatic animals that have been used for many years for their medicinal and ornamental properties. Annually, tens of millions of seahorses are collected from the wild and enter trade globally, mostly used for medicinal purposes [1–4]. Being scarce in population, seahorses are listed in the International Union for Conservation of Nature (IUCN) Red List of Threatened Species and the Convention on International Trade in Endangered Species of Wild Fauna and Flora (CITES) [5, 6]. The big-belly seahorse (*Hippocampus abdominalis*) is one of the largest seahorse species, and is mostly distributed in the temperate coastal regions of New Zealand and south-eastern Australia [6]. Owing to its high market value, the big-belly seahorse has become one of the major mariculture species in China in recent years, although full of challenges. One of the major threats to seahorse cultivation is frequent outbreaks of diseases, causing heavy economic losses.

Nervous necrosis viruses (NNVs) are non-enveloped positive-sense RNA viruses classified in the family *Nodaviridae*, which contains two genera: *betanodaviruses*, which predominantly infect fish, and *alphanodaviruses*, which predominantly infect insects [7]. *Betanodaviruses*-infected fish show abnormal swimming behaviour, such as spiraling and darting, due to vacuolation and necrosis of the central nervous system [8], furthermore, NNVs result in high mortality in hatchery-reared larvae and juveniles of a wide range of marine fish species in Asia, Europe, Australia, North America and North Africa [9–16]. The genome of NNV, packed into a diameter of 25–30 nm icosahedral virion, is comprised of two positive-sense RNA molecules: RNA1 encodes the RNA-dependent RNA polymerase (RdRp) required for viral genome replication, and RNA2 encodes the capsid protein (Cp), which is the single major structural protein of NNV and determines the host specificity [9, 17, 18]. According to the International Committee on Taxonomy of Viruses, NNVs are classified into four genotypes: striped jack NNV (SJNNV), tiger puffer NNV (TPNNV), barfin flounder NNV (BFNNV), and red-spotted grouper NNV (RGNNV) [18]. Highly contagious and virulent, NNV of all genotypes has been identified in more than 40 species of marine and freshwater fish, either asymptomatic or causing neural disease [9, 19]. However, no NNV infected seahorses have been recorded.

Recently, we have observed a disease causing spiral swim pattern, abdominal distension and high lethality in farmed seahorses in Fujian, China, without parasitic infection, bacterial infection, mycotic infection, external or organ injuries, or water pollution. It was highly suspected that the seahorses were infected with viruses, and we analysed the pathogens in this work. Viruses that affected seahorses are scarcely studied, partially because of the restricted seahorse-aquaculture scale, and the lack of research on this species. Our study is the first identification of a virus inducing VNN in seahorses, and we nominate it seahorse NNV (SHNNV). We report the isolation, characterization and pathogenicity of a SHNNV isolate from the big-belly seahorse. Although many studies of NNVs have been conducted, nowadays, there are still limited methods of preventing NNVs’ invasion of fishes, for the small scale application of effective drugs or vaccines. The isolation and identification of SHNNV will contribute to the research on both pathogenesis and therapeutic methods of NNV. In summary, the present study provides a basic resource for the subsequent investigation of big-belly seahorse and SHNNV interactions, as well as a new species applicable for NNV studies.

## Methods

### Sample collection

In this study, diseased big-belly seahorses (average weight of 0.33 ± 0.12 g, average body length of 7.21 ± 1.03 cm) were obtained from a fish farm in Xiamen city, Fujian Province, China. The fish showed abnormal swimming behaviour, and the cumulative mortality rate was approximately 40% in one month. Eye, brain, rhynchodaeum, liver, intestine, brood pouch, bone, muscle, gonad, heart, kidney, gill, skin, and gallbladder tissues of each fish were analysed individually for the presence of NNVs.

### Viral isolation

Eye and brain tissue samples of NNV-positive seahorses were homogenized in 5 mL of L15 medium (Gibco, USA) without foetal bovine serum (FBS). Then, the tissue homogenates were filtered through a 0.22 μm filter membrane, inoculated on grouper spleen (GS) cells, and cultured in L15 medium supplemented with 10% FBS (Gibco, USA). The same volume of L15 was added to the mock infected group. Inoculated cells were cultured at 28 °C and monitored regularly for the development of cytopathic effects (CPEs). The virus isolate was propagated in GS cells until the cell monolayer was destroyed. The cell culture supernatant was then recovered, centrifuged at 1,000 × g for 10 min at 4 °C and stored at − 80 °C until use. Viral titres were determined by the 50% tissue culture infective dose (TCID50) method.

### Identification of the isolated viruses by PCR

Total RNA was extracted from the supernatants of CPE-positive cultures using an RNA extraction kit (Takara, Japan) and reverse transcribed using a Transcription First Strand cDNA Synthesis Kit (Roche, Switzerland) according to the manufacturer’s instructions. For the detection of SHNNV, PCR was performed using Blend Taq DNA polymerase (Toyobo, Japan) with the following conditions: denaturation at 94 °C for 2 min, followed by 35 cycles of 94 °C for 30 s, 55 °C for 30 s, and 72 °C for 90 s. The reactions were completed with a final extension of 10 min at 72 °C. The PCR product was analysed by electrophoresis on a 1.5% agarose gel in TAE buffer containing GoldView I nucleic acid stain (Solarbio, China). The primers used are listed in Table 1. PCR products were cloned into PMD-18 T vectors (Takara, Japan) and sequenced by a sequencing company (BGI, China).

**Table 1:** Primers used in this study

*Primers used in gene cloning*	
cp-F:CACCGCTTTGCAATCACAATG	cp-R:GTCATCAACGATACGCACTAGG
*Primers used in RT-qPCR*	
q-cp-F:GATACGCTGTTGAAACACTGG	q-cp-F:GGAACGCTCAGTCGAACACTC
q-β-actin-F: ACCATCGGCAATGAGAGGTT	q-β-actin-R: ACATCTGCTGGAAGGTGGAC

### Sequence alignment analysis

The putative CP amino acid sequences were predicted using BioEdit software [20]. Phylogenetic analysis based on the protein sequence was performed with MEGA 6.0 using the neighbour joining method with resampling with 1000 bootstrap replicates [21].

### Tissue distribution of viruses

To investigate which tissues the virus mainly infects, total RNA was extracted from different tissues of diseased big-belly seahorses, including the eye, brain, rhynchodaeum, liver, intestines, brood pouch, bone,muscle, gonad, heart, kidney, gill, skin, and gallbladder. Transcripts of *cp* gene from different tissues were examined by quantitative real-time PCR (qRT-PCR). The qRT-PCR conditions were as follows: denaturation at 94 °C for 1 min, followed by 40 cycles of 94 °C for 15 s, 55 °C for 15 s, and 72 °C for 60 s. Standard amplification curves for different genes were generated via serial dilutions of plasmid constructs. The concentration of the template in the samples was determined by relating the Cq value to the standard curve. The β-actin gene was used as an internal control. The primers used in this study are listed in Table 1.

### *Fluorescence *in situ* hybridization (FISH)*

Sense and antisense digoxigenin (DIG)-labelled riboprobes were synthesized from the open reading frame sequence of the *cp* gene using a DIG RNA Labelling Kit (Roche Diagnostics, Germany).

The procedures for RNA FISH followed those of Ragoczy et al. [22], Ho et al. [23], and Beliveau et al. [24], with modifications. Briefly, NNV-positive seahorses were fixed in buffered 4% paraformaldehyde for 24 h. The samples were then dehydrated with a series of graded ethanol solutions (70–100%), cleared in xylene and embedded in paraffin. Ten-micron sections were cut for FISH. Prior to hybridization, the slides were washed with phosphate-buffered saline (PBS), sequentially dehydrated in 70%, 90%, and 100% ethanol, and equilibrated in 10% formamide / 2 × saline sodium citrate(SSC), pH 7.0. A mixture of the primary sense and antisense probes and the secondary probes was hybridized to the cells in 10% formamide / 10% dextran sulfate / 2 × SSC / 5 mmol/L ribonucleotide vanadate complex / 0.05% bovine serum albumin / 1 μg/μL *E. coli* tRNA and hybridized overnight at 55 °C in a humidified chamber. Slides were sequentially washed in 10% formamide / 2 × SSC, pH 7.0, followed by 2 × SSC at 37 °C and then mounted with Fluoroshield with DAPI (Sigma, USA). Fluorescence signals from FISH were imaged with a Zeiss confocal microscope (Germany).

### Brain and eye histology

The brains and eyes were removed from diseased and healthy big-belly seahorses, fixed in Bouin’s solution overnight at room temperature, dehydrated, and then embedded in paraffin wax. All tissue blocks were sectioned at a thickness of 5 μm and stained with hematoxylin and eosin for subsequent analysis.

### Infection experiment

To investigate the pathogenicity of SHNNV, juvenile big-belly seahorses were experimentally infected with SHNNV. Juvenile big-belly seahorses (average body length of 7.89 ± 0.40 cm) were purchased from a fish farm and maintained at 21 ± 1 °C in tanks under a natural photoperiod. Juvenile big-belly seahorses were divided into two groups with 50 fish per group. A viral suspension was diluted tenfold, and each fish in the infection group received 30 μL diluted virus by intraperitoneal injection. The control group was injected with 30 μL PBS per seahorse. Seahorses were monitored twice daily for clinical signs of pathology and mortality.

### Statistical analysis

The results were analysed statistically using repeated measurements of variance analysis with SPSS software 25 [25].

## Results

### Isolation and identification

**Fig. 1:**
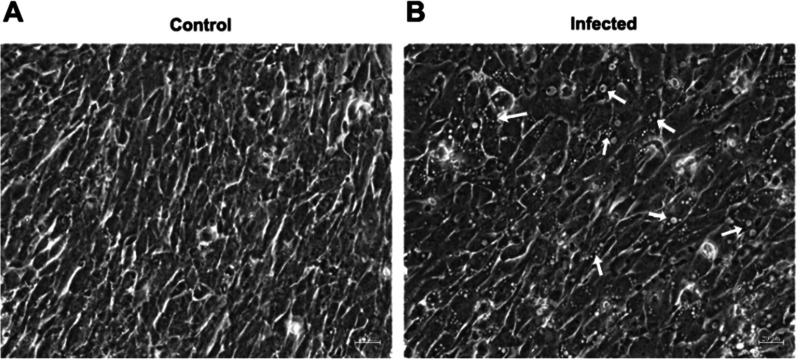
Detection of SHNNV by cell culture and PCR. GS cells were incubated with brain and eye homogenates of SHNNV-positive big-belly seahorses. CPEs were observed in the infected group (**B**). Uninfected GS cells were used as controls (**A**). The white arrow indicates the CPE

**Fig. 2:**
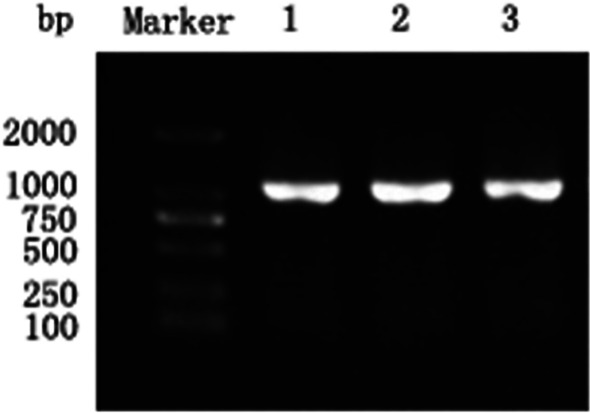
Agarose gel electrophoresis of PCR products from infected GS cells using specific primers for the SHNNV *cp* gene; lane 1 ~ 3: PCR products from infected GS cells

**Fig. 3:**
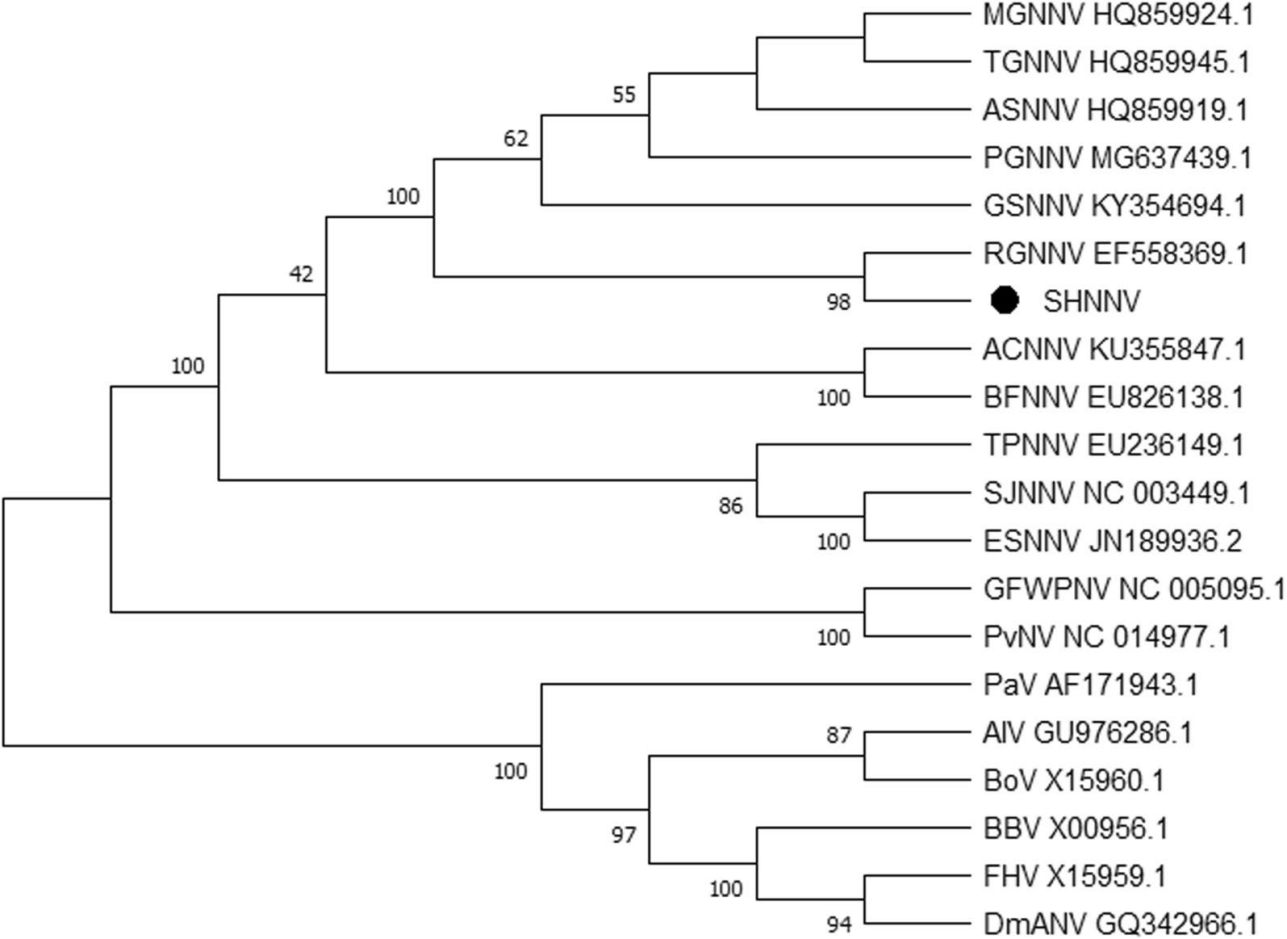
Phylogenetic analysis of SHNNV isolates. Phylogenetic trees based on amino acid sequences and the gene coding for CP. The numbers at the end of the virus species names denote the GenBank accession number. The phylogenetic tree was constructed using the neighbour‐joining methods with 1,000 non‐parametric boot‐strap replicates in MEGA 6.0

Brain and eye tissue homogenates of diseased big-belly seahorses were inoculated onto a GS cell monolayer at 28 °C. Significant CPEs were observed at 24 h in GS cells infected with virus (Fig. 1B). No CPE was observed in the control group (Fig. 1A). The infected cells were NNV-positive by PCR assays, and the PCR products of the estimated size (1073 bp) were identified, with clear electrophoretic bands (Fig. 2). The 1017 bp full-length gene encoded 338 amino acids (Additional file 1: Sequence S1 and S2). A phylogenetic tree was constructed by the neighbour-joining method with1000 bootstrap replicates based on a multiple alignment of the gene sequences, which were predicted using the standard genetic code. The phylogenetic tree showed that the virus clustered with NNV and was most closely related to RGNNV; therefore, the isolated virus strain was designated as SHNNV (Fig. 3).

### Tissues mainly infected with SHNNV

**Fig. 4:**
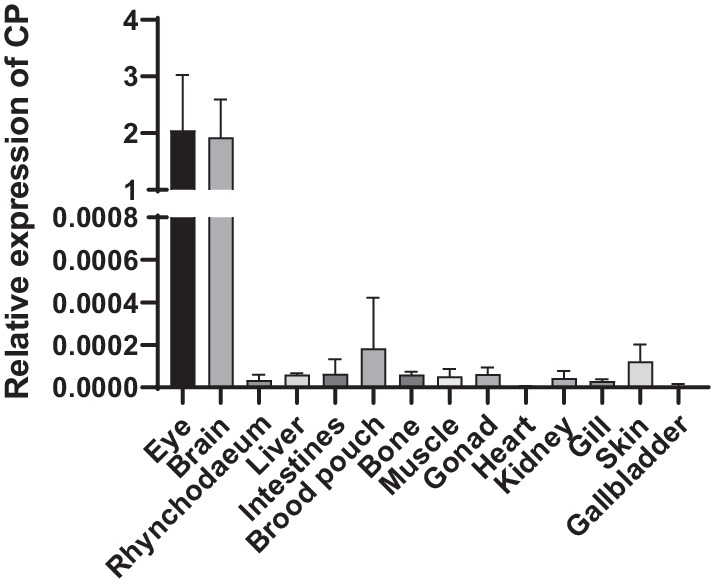
RT-PCR analysis of the expression patterns of the SHNNV *cp* gene in big-belly seahorses. β-actin was used as the house keeping gene control. Data are presented as the mean ± standard deviation (n = 3)

**Fig. 5:**
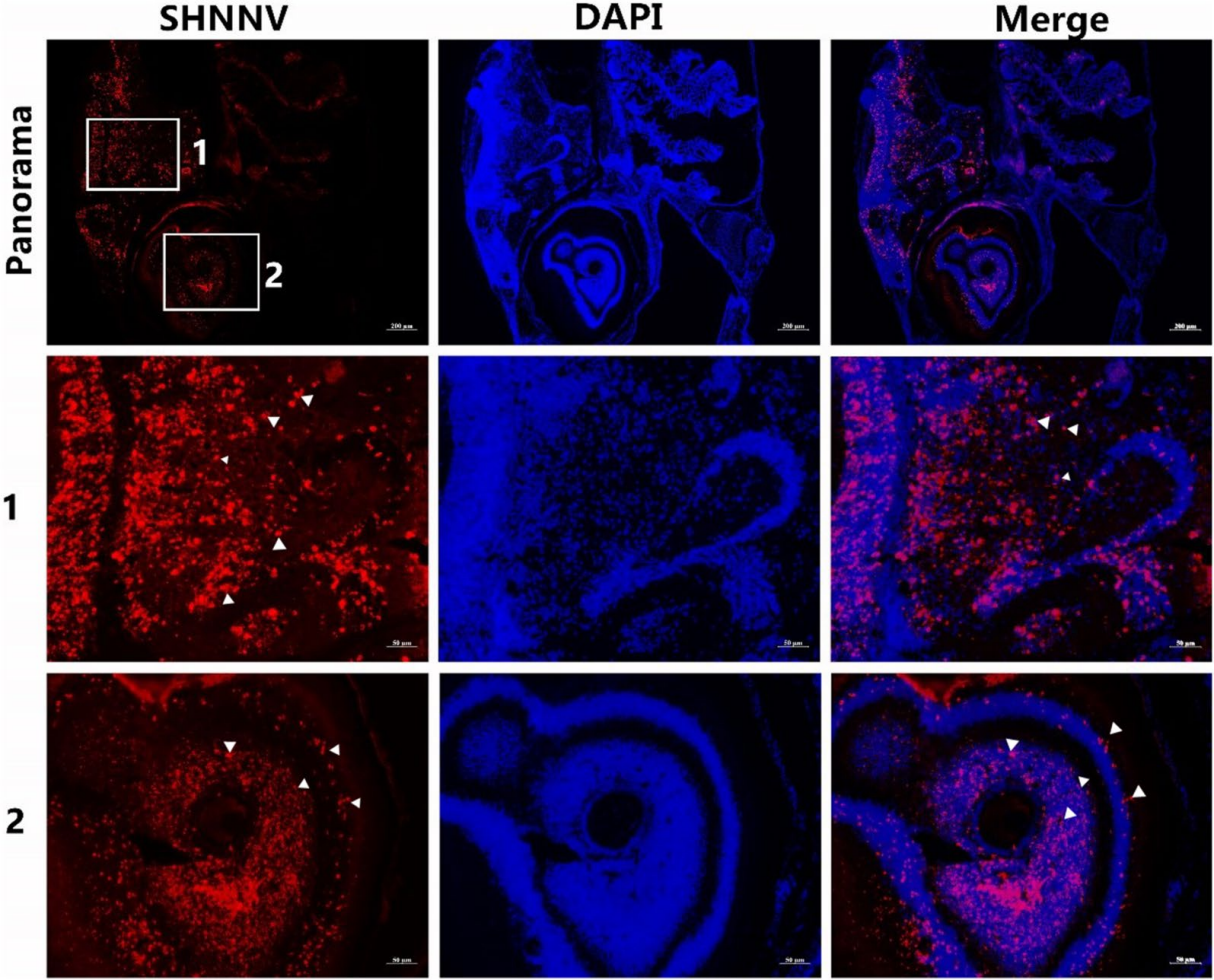
Main tissues attacked by SHNNV. Red staining indicates the Cp position. The middle row (1) shows high magnification of boxed area 1 in the top row (panorama). The bottom row (2) shows high magnification of boxed area 2 in the top row (panorama). SHNNV was mainly localized in the brain and eye. The white arrow points SHNNV

To investigate the target tissues of SHNNV, we examined the transcription level of *cp* mRNA in various tissues from diseased big-belly seahorses by qRT-PCR. As shown in Fig. 4, its transcriptional level was significantly increased in the brain and eye. The virus was also found in brood pouch, skin, muscle and other organs, but only with extremely low viral load. We further performed FISH of the *cp* mRNA. From the Fig. 5, we found the positive signals were mainly detected in the brain and retina which consistent with qRT-PCR result (Fig. 5). These results indicate that SHNNV mainly infects the brain and eye of big-belly seahorses.

### Histopathology of SHNNV infection

**Fig. 6:**
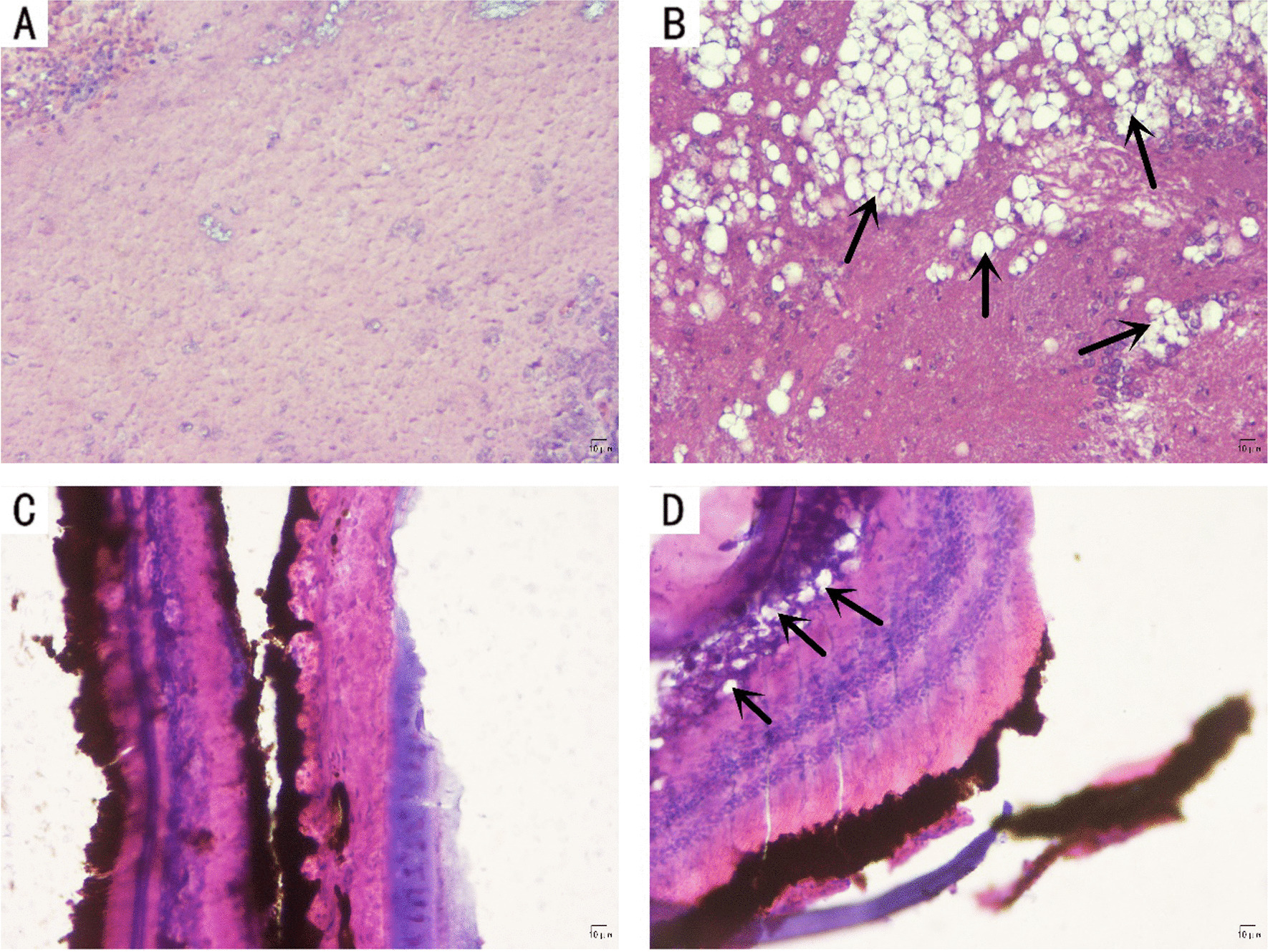
Micrographs of H&E-stained brain and eye tissues of big-belly seahorses naturally infected with SHNNV. **A** H&E staining of normal big-belly seahorse brain tissue. **B** H&E staining of infected big-belly seahorse brain tissue. Many cytoplasmic vacuolations (black arrows) were found in the brain tissue. **C** H&E staining of normal big-belly seahorse eye tissue. **D** H&E staining of infected big-belly seahorse eye tissue. Many cytoplasmic vacuolations (black arrows) were found in the ganglion cell layer

To investigate the pathology of big-belly seahorse brain and retinal tissue caused by SHNNV infection, we performed histological analysis on brain and retinal tissues of healthy and diseased seahorses. Compared to the healthy ones, SHNNV-positive seahorses showed cytoplasmic vacuolation in the brain (Fig. 6A, B) and retinal tissues (Fig. 6C, D).

### SHNNV isolate pathogenicity tests

**Fig. 7:**
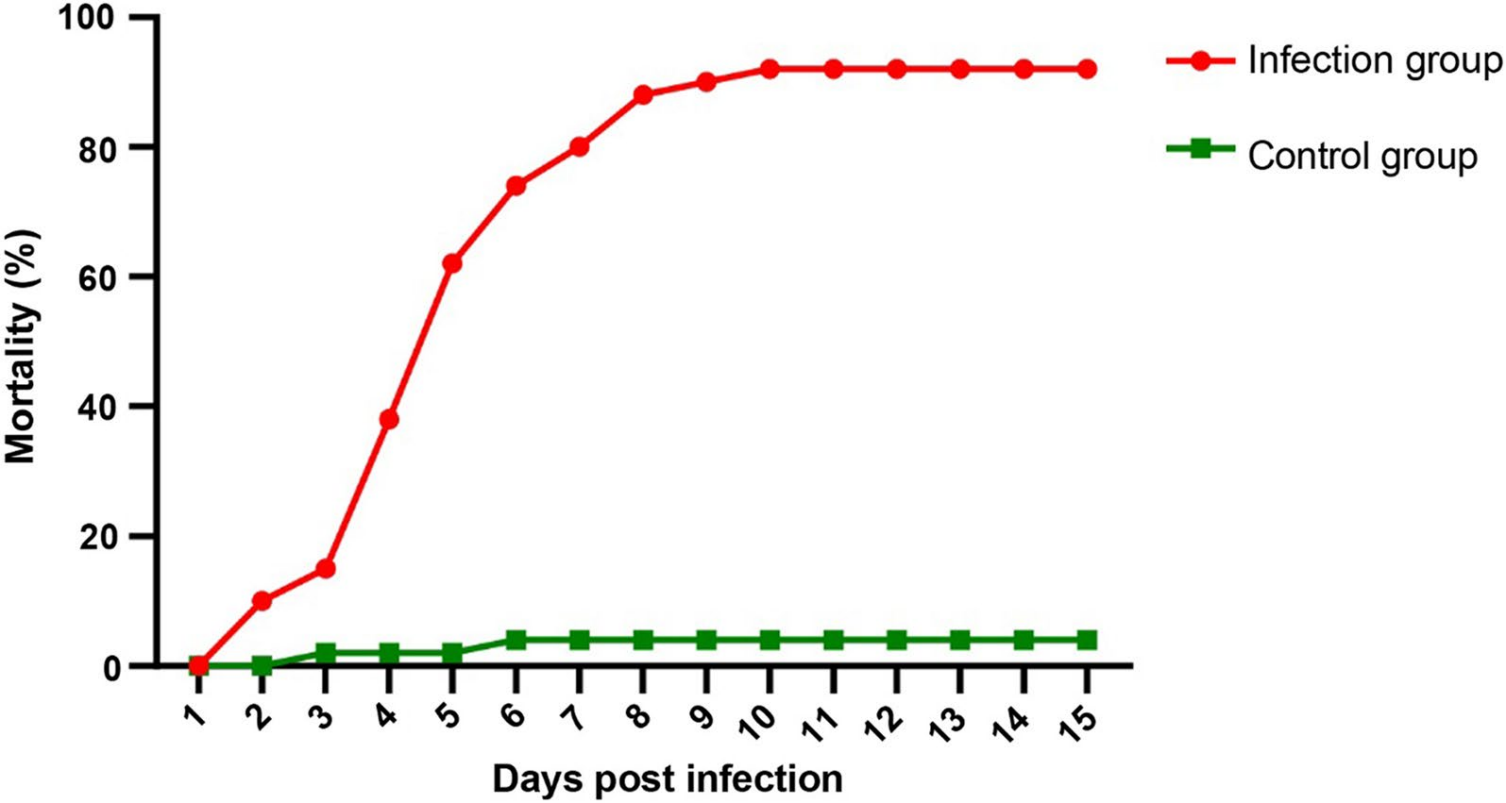
Cumulative mortality (%) of juvenile big-belly seahorses challenged with SHNNV. The cumulative mortality was determined from 1 to 15 days post infection

To evaluate the pathogenic potential of SHNNV in fish hosts, big-belly seahorses were infected with the virus by intraperitoneal injection. In the infection group, the seahorses began to die from the second day, and the survivors tended to stabilize over the ten days. The death rate was 92%. In the control group, only two seahorses died, with a mortality rate of 4% (Fig. 7).

## Discussion

In the present study, a virus strain was isolated from diseased big-belly seahorses in Xiamen city. By employing a series of biochemical and biophysical methods, we first identified that the causative agent was an SHNNV strain belonging to *betanodavirus*. The history of VNN researches can be traced back to the mid-1980s. In 1985, a new disease with a mortality rate greater than 90% occurred in Australia, Japan, Southeast Asia, and the Caribbean in almost simultaneous outbreaks in the breeding of a variety of young marine fish [10, 13, 26, 27]. In China, the first NNV isolation was reported in reared stone red-spotted grouper in 2001 [28]. Although some cases of similar symptoms have been discovered in numerous marine fish infected with NVV, no formal reports on seahorse nervous necrosis have been recorded. Thus, the first observation of a disease causing swimming in spirals or rotations, lethargy and anorexia in seahorses has not been reminiscent of NNV at the beginning, whereas further environmental and pathogenic analysis indicated virus infection. The typical symptom of VNN and the afterwards demonstrated vacuolation in the brain and eye confirmed that the causative agent belonged to *betanodavirus*. The pathogenic test showed acute viral infection and an extremely high case fatality rate of 92% compared to the control group (4%).

In previous studies, researchers commonly identified and classified NNV by analysing the sequences of RNA2 or the *cp* genes from various viral isolates [7, 14, 29, 30]. *Cp* gene encodes the capsid protein, which contains a conservative shell domain (S-domain), a hypervariable protrusion domain (P-domain) and an N-terminal arm recruiting genomic RNA [31]. It has been indicated that CP is the major determinant of immunoactivity and host specificity [17, 32], and partially determines the thermotolerance of NNV [33, 34]. Here we identified the virus by analysing the full length of *cp* mRNA amplified by primers that designed according to a known NNV isolate. Phylogenetic analysis indicated that SHNNV was most closely related to RGNNV genotype.

Previous studies have shown that RGNNV mainly destroys the brain and eyes of the infected fish, and the prominent pathological features are vacuolation of the brain and retina, resulting in massive losses to the population of grouper and other marine fishes [8, 35, 36]. It is reported that RGNNV also detected in gills, intestine, stomach, spleen, liver, kidney, pyloric gland, skeletal muscle, blood cells and fin [8, 9, 36]. In the present study, according to the results of the tissue distribution of the virus, brain and eye histology and FISH, we found that SHNNV mainly destroyed the big-belly seahorse brain and eye and caused vacuolation of the brain and retina. These results were consistent with the pathology of NNVs. We also detected a low expression level of viral mRNA in rhynchodaeum, liver, intestine, brood pouch, bone, muscle, gonad, heart, kidney, gill, skin, and gallbladder tissues.

In our unpublished study, we found that SHNNV was sensitive to water temperature. Many studies have discovered a temperature-dependent effect of NNV infection, with distinct optimum growth temperature in four genotypes. Unlike the other three genotypes that replicate in cold water, the optimum temperature for RGNNV replication was 25–28 °C, corresponding to the water temperature of VNN outbreaks [34, 37–41]. The temperature range for RGNNV genotype, however, has not yet been definitely verified, while temperature that too high or too low was unsuitable for host cells to grow [42]. In our study, the seahorses were raised at 21 °C, and our further research showed that SHNNV could replicate at 13 °C, the temperature was far below the temperature optima, and below the lowest experimental temperature record [33, 42, 43]. The seahorse *Hippocampus abdominalis* can be cultured as low as 8 °C, and compared to other fish, it is small, rapid-propagative and easy to culture in experimental conditions, giving it an advantage for NNV researches.

A research showed a distinct difference between the RNA2 sequences of RGNNV isolates in fish held at high temperature and low temperature [44]. However, it is also reported that RNA1, encoding RdRp, protein B1 and B2, is the key contributing factor for the response to temperature [33], Ciulli et al*.* also considered that RNA1 rather than RNA 2 plays a more important part in thermotolerance of NNV, although RNA2 was found to determine temperature sensitivity as well [34, 43]. There is evidence that the RNA1 evolved rapidly than RNA2, indicating a strong selection to respond to various temperature ranges [15, 45, 46]. In our study, we only analysed the *cp* gene but not the complete sequence of RNA1 and RNA2. The next plan of analysing the genetic divergence of between SHNNV and other RGNNV isolates will be supplement to the studies of the thermal adaptability of NNV.

## Conclusions

In conclusion, we isolated and characterized SHNNV from reared big-belly seahorses in China. The present isolate was closely related to known RGNNV isolates. It can infect GS cells and induce obvious CPE. We also found that SHNNV mainly destructs the big-belly seahorse brain and eye and causes vacuolation of the brain and retina. The infection experiment revealed that SHNNV was virulent to juvenile big-belly seahorses. Further studies will focus on the infection mechanism and preventive strategies for SHNNV.

## Supplementary Information

**Additional file1.** **Sequence S1.** Nucleotide sequence of SHNNV-cp gene; **Sequence S2.** Amino acid sequence deduced from SHNNV-cp mRNA.

## Data Availability

Nucleotide and amino acid sequences supporting reported results can be found in the Additional file 1: All data are presented in the results section and expressed into graphic presentations. If any data, not already shared, are needed, they may be made available.

## Abbreviations

BFNNV: Barfin flounder NNV
CITES: Convention on International Trade in Endangered Species of Wild Fauna and Flora Conservation programs
CP: Capsid protein
CPE: Cytopathic effects
DIG: Digoxigenin
FBS: Fetal bovine serum
FISH: Fluorescent in situ hybridization
GS: Grouper spleen
IUCN: International Union for Conservation of Nature
NNV: Nervous necrosis virus
qRT-PCR: Quantitative real-time PCR
RdRp: RNA-dependent RNA polymerase
RGNNV: Red-spotted grouper NNV
SHNNV: Seahorse nervous necrosis virus
SJNNV: Striped jack NNV
SSC: Saline sodium citrate
TCID50: Median tissue culture infective dose
TPNNV: Tiger puffer NNV
VNN: Viral nervous necrosis
