# Supplementary material for: Isolation and identification of a new strain of nervous necrosis virus from the big-belly seahorse Hippocampus abdominalis
Source: Virol J. 2022 Jun 27;19:109. doi: 10.1186/s12985-022-01837-8 (PMC9235245; doi:10.1186/s12985-022-01837-8)
Supplement: Supplementary file 1 — Additional file1. Sequence S1. Nucleotide sequence of SHNNV-cp gene; Sequence S2. Amino acid sequence deduced from SHNNV-cp mRNA. [file 12985_2022_1837_MOESM1_ESM.pdf]

Isolation and identification of a new strain of nervous necrosis virus from the big-belly seahorse *Hippocampus abdominalis* in China

Supplementary material

Sequence S1. Nucleotide sequence of SHNNV-*cp* gene

ATGGTACGCAAAGGTGAGAAGAAATTGGCAAAACACGCGACCACCAAGGCCGCGAAT
CCGCAACCCCGCCGACGTGCTAACAATCGTCGGCGTAGTAACCGCACTGACGCACCTG
TCTCTAAGGCCTCGACTGTGACTGGATTTGGACGTGGGACCAATGACGTCCATCTCGC
AGGTATGTCGAGAATCTCCCAGGCCGTCCTCCCAGCCGGGACAGGAACAGACGGATAC
GTTGTTGTTGACGCAACCATCGTTCCCGACCTCCTGCCACGACTGGGACACGCTGCTA
GAATCTTCCAGCGATACGCTGTTGAAACACTGGAGTTTGAAATTCAGCCAATGTGCCC
CGCAAACACGGGCGGTGGTTACGTTGCTGGCTTCCTGCCTGATCCAACTGACAACGAT
CACACCTTCGACGCGCTTCAAGCAACTCGTGGTGCAGTCGTTGCCAAATGGTGGGAA
AGCAGAACAGTCCGACCTCAGTACACCCGCACGCTCCTCTGGACCTCGTCGGGAAAG
GAGCAGCGTCTCACGTCACCTGGTCGGCTGATACTCCTGTGTGTCGGCAACAACACTG
ACGTGGTCAACGTGTCGGTGCTGTGTCGCTGGAGTGTTCGACTGAGCGTTCCATCTCT
TGAGACACCTGAAGAGACCACCGCTCCCACCATGACACAAGGTTCCCTGTACAACGAT
TCCCTTTCCACAAATGACTTCAAGTCCATCCTCCTAGGATCCACGCCACTGGACATTGC
CCCTGATGGAGCAGTCTTCCAGCTGGACCGTCCGCTGTCCATCGACTACAGCCTTGGA
ACTGGAGATGTTGACCGTGCTGTTTACTGGCACCTCAAGAAGTTTGCTGGAAATGCTG
GCACACCTGCAGGCTGGTTTCGCTGGGGCATCTGGGACAACTTCAACAAGACGTTCAC
AGATGGCGTTGCTTACTACTCTGATGAGCAGCCCCGTCAAATCCTGCTGCCTGTTGGCA
CTGTCTGCACCAGGGTTGACTCGGAAAACTAA

Sequence S2. Amino acid sequence deduced from SHNNV-*cp* mRNA

MVRKGEKKLAKHATTKAANPQPRRRANNRRRSNRTDAPVSKASTVTGFGRGTNDVHLA
GMSRISQAVLPAGTGTDGYVVVDATIVPDLLPRLGHAARIFQRYAVETLEFEIQPMCPANT
GGGYVAGFLPDPTDNDHTFDALQATRGAVVAKWWESRTVRPQYTRTLLWTSSGKEQRLT
SPGRLILLCVGNNTDVVNVSVLCRWSVRLSVPSLETPEETTAPTMTQGSLYNDSLSTNDFK
SILLGSTPLDIAPDGAVFQLDRPLSIDYSLGTGDVDRAVYWHLKKFAGNAGTPAGWFRWG
IWDNFNKTFTDGVAYYSDEQPRQILLPVGTVCTRVDSEN*
